# The impact of implementation of rapid blood culture identification panels on antimicrobial optimization: a retrospective cohort study

**DOI:** 10.1017/ash.2024.51

**Published:** 2024-04-16

**Authors:** Tyler Martin, Eli Wilber, Shreena Advani, Joseph Torrisi, Manish Patel, Paulina A. Rebolledo, Yun F. Wang, Sheetal Kandiah

**Affiliations:** 1 Department of Pharmacy, Grady Health System, Atlanta, GA, USA; 2 Division of Infectious Diseases, Department of Medicine, Emory University School of Medicine, Atlanta, GA, USA; 3 Hubert Department of Global Health, Rollins School of Public Health, Emory University, Atlanta, GA, USA; 4 Grady Healthcare System, Atlanta, GA, USA; 5 Clinical Microbiology Laboratory, Grady Health System, Atlanta, GA, USA; 6 Department of Pathology and Laboratory Medicine, Emory University School of Medicine, Atlanta, GA, USA

## Abstract

**Objective:**

Determine the impact of limited implementation of a rapid blood culture identification (BCID) panel.

**Design:**

Retrospective cohort study.

**Methods:**

From February to April 2022, positive blood cultures identified via e-Plex BCID (Roche, Carlsbad, CA) were compared to those identified using standard microbial identification techniques. The primary outcomes assessed were time to optimal therapy, time to de-escalation of anti-MRSA (methicillin-resistant Staphylococcus aureus) agents, and time to de-escalation of anti-pseudomonal agents. Additional analysis investigated the impact of the availability of antimicrobial stewardship program support. This study was conducted at Grady Health System, a large metropolitan safety-net hospital in the southeastern United States.

**Results:**

A total of 253 blood cultures were included in this study (153 BCID and 100 standard). Blood culture identification use was associated with a reduction in median time to optimal antimicrobial therapy (43.4 vs 72.1 h, *P* < .001) and median time to de-escalation of anti-MRSA agents (27.7 vs 46.7 h, *P* = .006), and a trend towards reduction of median time to de-escalation of anti-pseudomonal agents (38.8 vs 54.8 h, *P* = .07). These reductions persisted when controlling for patient age, sex, intensive care unit status, Charlson Comorbidity Index, and antimicrobial stewardship program availability.

**Conclusions:**

Despite restricted use and lack of 24/7 antimicrobial stewardship program availability, BCID panel utilization was associated with earlier initiation of optimal therapy and pathogen identification with subsequent de-escalation of broad-spectrum antimicrobials, as compared to standard antimicrobial techniques. This suggests the potential for benefit from adopting novel diagnostic technologies outside of idealized fully-resourced settings.

## Introduction

Blood culture identification (BCID) panels utilize a polymerase chain reaction-based approach to identify bacterial and fungal pathogens from positive blood cultures.^1^ This allows for rapid organism identification in the electronic medical record after blood culture positivity. Additionally, BCID panels detect the presence of various antibiotic resistance genes, enabling the optimization of antimicrobial therapy sooner than standard culture-based techniques, which typically take 48–72 hours.^2^

Several studies have shown rapid pathogen identification, in conjunction with antimicrobial stewardship interventions, can improve time to effective therapy, reduce broad-spectrum antibiotic use, and decrease hospital length of stay.^2–5^ Moreover, an economic evaluation of BCID implementation reported identification of coagulase-negative *Staphylococci* (CoNS) contaminants resulted in approximately $30,000 in savings per 100 patients tested.^6^

Grady Health System recently implemented e-Plex BCID (Roche, Carlsbad, CA) for identification of positive blood cultures. Due to reagent and staffing limitations, BCID was only conducted on cultures turning positive during day shift in the microbiology lab (7 A.M. to 5 P.M., seven days a week). The antimicrobial stewardship program (ASP) provides real-time audit and feedback during usual business hours (7 A.M. to 5 P.M., Monday through Friday) for all positive blood cultures identified through both BCID and standard microbiological techniques.

This model of BCID implementation provides an opportunity to evaluate the impact of BCID while controlling for variation in provider practice patterns, as the same providers would be caring for bacteremic patients with and without BCID results. This contrasts with previous studies where BCID technology was utilized on all positive blood cultures and was typically compared to historical controls. This study aims to leverage this opportunity to evaluate the impact of BCID implementation on time to optimal antimicrobial therapy and de-escalation of broad-spectrum antimicrobials. Additionally, this restricted model of BCID implementation has not previously been assessed and may be of interest to institutions with similar staffing limitations, which were exacerbated by, and have persisted after, the COVID-19 pandemic. We hypothesized that cultures analyzed with BCID would have a shorter time to optimal therapy and de-escalation of broad-spectrum agents.

## Methods

### Study design

This was a single-center, retrospective cohort study at a 953-inpatient-bed academic medical center in metropolitan Atlanta, Georgia. All positive blood cultures from adult patients between February 1 and April 30, 2022 were included. Exclusion criteria included discordant results between BCID and standard identification techniques from the same bottle, growth of the same species within five days prior to the index culture, initiation of antimicrobials ≥48 hours prior to blood culture collection, lack of antimicrobial administration, patient death/discharge/transfer within 24 hours of blood culture collection, and growth of an organism not identifiable by BCID. Multiple blood cultures drawn from the same patient were included if criteria were met. Table S1 contains the organisms and resistance genes identifiable by each BCID panel.

Positive blood cultures over the study period were identified via an electronic infection surveillance system (VigiLanz Corporation, Chicago, IL). Clinical, demographic, and microbiologic data were extracted from the electronic medical record. Two investigators independently reviewed each case to determine optimal antimicrobial therapy, and time to optimal antimicrobial therapy, and to identify cases where multiple potential infections were being treated. Differences were resolved through discussion. This study was approved by the Emory Institutional Review Board and Grady Research Oversight Committee.

### Blood culture methods

Blood cultures are incubated and monitored using the BacT/ALERT VIRTUO system (bioMérieux Inc., Marcy-l'Étoile, France). Positive blood cultures undergo a Gram stain and plating to solid media with real-time notification of Gram stain results to clinicians as a critical result. Colonies from solid media are identified using matrix-assisted laser desorption/ionization-time of flight mass spectrometry (VITEK MS, bioMérieux). Antimicrobial susceptibility testing is performed by VITEK2 (bioMérieux) serial broth dilution. Additionally, BCID panels were performed on the first positive bottle for cultures turning positive between 7 AM and 5 PM. BCID is not repeated on the same patient within five days of the original BCID unless a different Gram stain result is found. Blood culture identification results are uploaded to the electronic medical record without a second critical result notification to clinicians and are accompanied by templated interpretation guidance drafted by the microbiology laboratory, ASP, and other infectious diseases clinicians. From 7 A.M. to 5 P.M. Monday through Friday, the ASP monitors all positive blood cultures and BCID results via VigiLanz to provide real-time treatment guidance.

### Outcomes

The primary outcomes of interest included time to optimal antimicrobial therapy, time to de-escalation of anti-MRSA (methicillin-resistant *Staphylococcus aureus*) agents, and time to de-escalation of anti-PsA (*Pseudomonas aeruginosa*) agents. Time to optimal therapy was defined as the time in hours from blood culture collection to initiation of the antimicrobial agent and dose expected to result in the best outcomes as determined by independent review of two study investigators (TM, EW). In cases where multiple antimicrobial agents had activity, the most narrow-spectrum agent was considered optimal. For example, ceftriaxone would be considered optimal therapy for a sensitive Enterobacterales organism, although piperacillin-tazobactam or cefepime would also be effective. Disagreements between the two independent reviewers on selection of optimal therapy were adjudicated by a third infectious diseases clinician (SK) on a case-by-case basis. Time to de-escalation was defined as the time in hours from administration of the first dose to the time of agent discontinuation.

Time to optimal therapy was not assessed when the optimal agent was initiated empirically, as this would confound results. For patients who did not receive optimal therapy, the full duration of the antimicrobial was considered as the time to optimal therapy. Any antibiotics received upon discharge for the treatment of the blood culture were included in the time to optimal therapy analysis. Time to de-escalation of anti-MRSA and anti-PsA agents was not assessed for patients with respective growth of MRSA or PsA, for those whose optimal therapy agent had activity towards MRSA or PsA, or for those who were not initiated on an anti-MRSA or anti-PsA agent, respectively.

Secondary outcomes included time to organism identification, vancomycin days of therapy (DOT) for CoNS growing in one of two blood cultures, all-cause hospital mortality, and hospital length of stay. Growth of CoNS (or other common skin flora including *Bacillus*, *Corynebacterium, Cutibacterium acnes, Lactobacillus,* and *Micrococcus* species) in only one of two blood cultures was regarded as a contaminant, while growth in two or more blood cultures was considered a true infection.

Vancomycin DOT was not assessed for cultures with growth of methicillin-resistant CoNS in at least two blood cultures, as this was regarded as a true infection being treated with optimal therapy. Additionally, respective outcomes were excluded for events where de-escalation or initiation of optimal antimicrobial therapy occurred prior to the Gram stain results being reported in the electronic medical record.

In addition to the primary analysis, a subgroup analysis was conducted where cases in which antibiotics were clearly directed at an additional separate infection from the index bacteremia were excluded (ie patients with polymicrobial pneumonia).

### Statistical analysis

All statistical analyses were completed with Microsoft Excel for Microsoft 365 MSO (Version 2202 Build 16.0.14931.20128) or RStudio software (R version 4.1.2, R Foundation for Statistical Computing, Vienna, Austria). Two-tailed Mann-Whitney *U* tests were performed for continuous data outcomes and two-tailed *Z* tests for equality of proportions were performed for categorical data outcomes. To further test the impact of BCID on the primary outcomes, a multiple linear regression model was fit using patient age, sex, ICU (intensive care unit) status, Charlson comorbidity index, and whether the final result occurred during a time with ASP support as covariates. Outliers were identified by a Cook’s distance >3 times the mean and were excluded.

## Results

### Patient demographics

**Figure 1.**
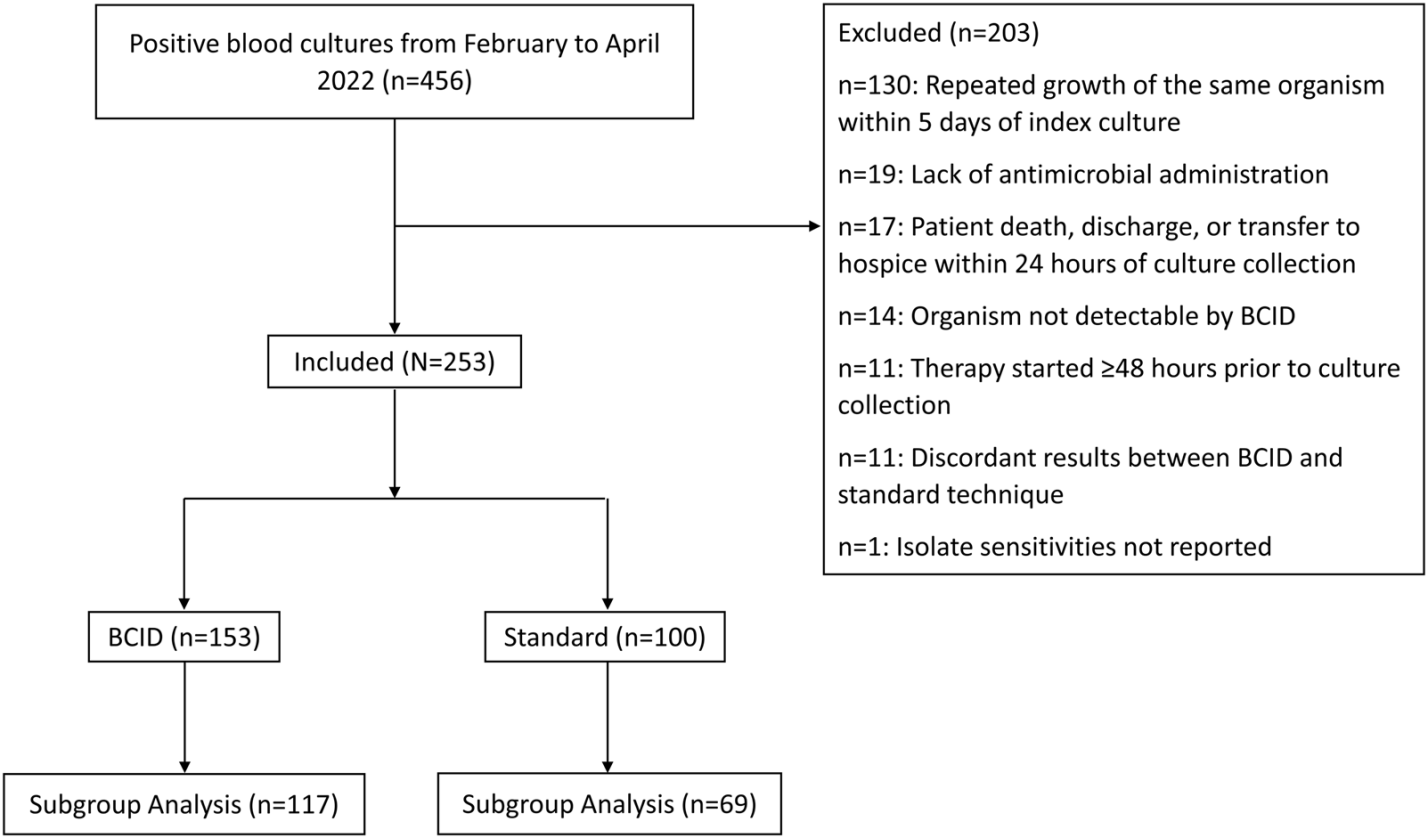
Study flow diagram of the included and excluded positive blood cultures. The subgroup analysis excluded patients receiving antimicrobials targeted at a different infection site.

456 positive blood cultures were identified within the study period. After applying the exclusion criteria, 253 remained (153 and 100 identified via BCID and standard techniques respectively). The most common cause of exclusion was repeated growth of the same organism within 5 days (Figure 1). Discordance between BCID and standard technique results occurred in 11 cases that were excluded (Table S2).

Baseline patient characteristics are summarized in Table 1. The BCID and standard technique groups were similar in age, gender, ethnicity, weight, Charlson comorbidity index, and proportion of patients in the ICU. The most frequently encountered organisms were CoNS, *Staphylococcus aureus*, *Streptococcus spp., E. coli,* and *Klebsiella spp.* Extended-spectrum beta-lactamase (ESBL)-producers were detected in 7 blood cultures, and fungi in 6. Vancomycin-resistant *Enterococci* and carbapenem-resistant organisms were not isolated.

**Table 1.** Baseline characteristics of the standard identification and blood culture identification groups

Baseline characteristic	Standard n = 100	BCID n = 153
Age in years, mean (SD)	59.6 (14.6)	59.1 (17.0)
Male, n (%)	67 (67)	90 (58.8)
Race/ethnicity, n (%)		
Black	82 (82)	116 (75.8)
White	11 (11)	21 (13.7)
Hispanic	4 (4)	9 (5.9)
Asian	1 (1)	2 (1.3)
Unknown	1 (1)	5 (3.3)
Multi-racial	1 (1)	0
Weight in kg, mean (SD)	80.3 (22.1)	76.8 (21.4)
Transfer to ICU, n (%)	49 (49)	83 (54.3)
Charlson comorbidity index, mean (SD)	4.7 (3.2)	4.6 (2.9)
Organism, n (%)		
CoNS	36 (36)	53 (34.6)
* Staphylococcus aureus*	20 (20)	41 (26.8)
MRSA	9 (45)	13 (31.7)
Enterobacterales	21 (21)	29 (19.0)
ESBL detected	4 (19)	3 (10.3)
* Streptococcus spp.*	11 (11)	19 (12.4)
* E. faecalis*	1 (1)	5 (3.3)
* Candida spp.*	2 (2)	4 (2.6)
* Pseudomonas aeruginosa*	4 (4)	3 (2.0)
Other	8 (8)	10 (6.5)
Polymicrobial	6 (6)	17 (11.1)

Note. Abbreviations; SD, standard deviation; kg, kilograms; ICU, intensive care unit; CoNS, coagulase-negative *Staphylococcus* species; MRSA, methicillin-resistant *Staphylococcus aureus*; ESBL, extended-spectrum beta-lactamase

### Outcomes

**Figure 2.**
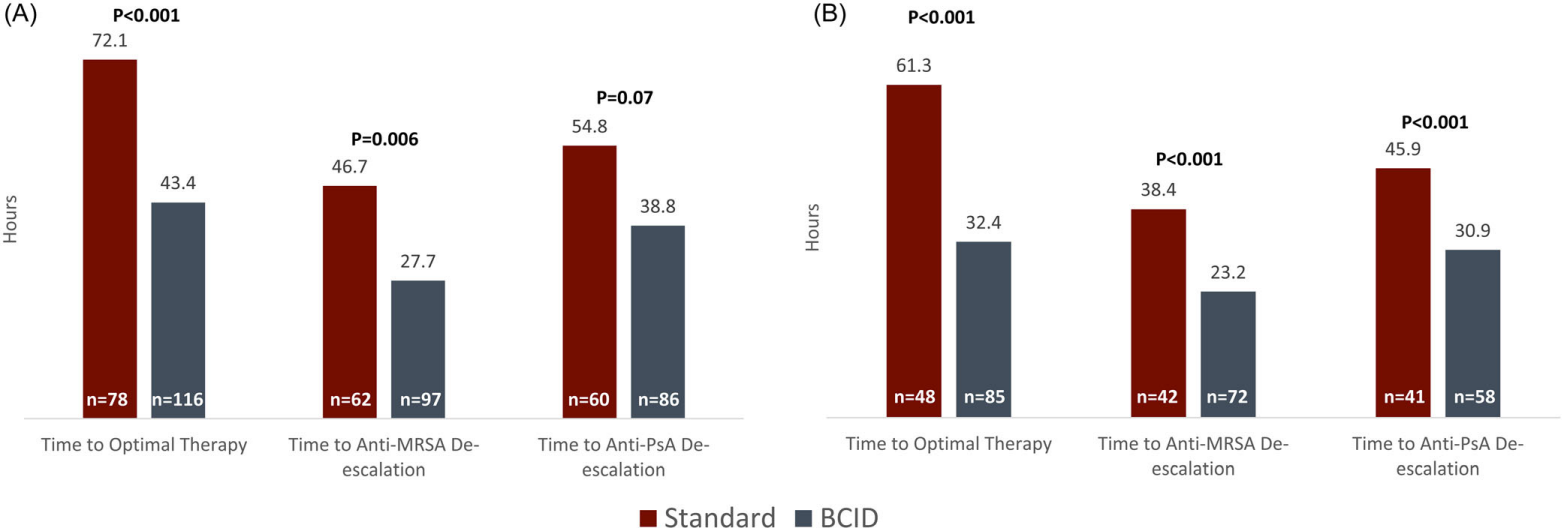
Primary outcomes of the primary and subgroup analyses. Primary outcomes of the primary analysis (Panel A) and the subgroup analysis (Panel B) with associated p-values. The subgroup analysis excluded patients receiving antimicrobials targeted at a different infection site.

In the primary analysis (Figure 2), use of BCID was associated with a significant reduction in median time to optimal therapy (43.4 h vs 72.1 h, difference –28.7 h, *P* < .001) and median time to anti-MRSA agent de-escalation (27.7 h vs 46.7 h, difference –19.0, *P* = .006) compared to standard microbial techniques. Use of BCID was associated with a trend towards a reduction in median time to anti-PsA agent de-escalation (38.8 h vs 54.8 h, difference –16.0 h, *P* = .07).

Regarding secondary outcomes, BCID use was associated with a significantly reduced median time to organism identification (23.9 h vs 45.3 h, difference –21.4 h, *P* < .001). There was no statistical difference in median vancomycin DOT for CoNS regarded as contaminants (3 d vs 4 d, *P* = .45), all-cause hospital mortality (10.5% vs 9.0%, *P* = .70), or median hospital length of stay (17.4 d vs 14.4 d, *P* = .42). These data are summarized in Table 2. During times without ASP support, BCID use was associated with a significant reduction in time to optimal therapy (47.9 h vs 75.6 h, *P* = .004) when compared to standard techniques. Among isolates identified using BCID, ASP support did not significantly reduce time to optimal therapy (39.5 h vs 47.9 h, *P* = .18), suggesting BCID panels may be a useful stewardship tool even in the absence of a robust ASP.

**Table 2.** Secondary outcomes of the primary and subgroup analyses

	Primary analysis			Subgroup analysis		
	Standard n = 100	BCID n = 153	*P*-value	Standard n = 69	BCID n = 117	*P*-value
Time to organism identification in hours, median (IQR)	45.3 (29.7–56.2)	23.9 (19.5–32.0)	<.001	37.4 (27.6–50.0)	23.0 (17.7–28.5)	<.001
All-cause hospital mortality, n (%)	9 (9)	16 (10.5)	.70	8 (11.6)	11 (9.4)	.63
Hospital length of stay in days, median (IQR)	14.4 (6.6–33.9)	17.4 (8.1–33.2)	.42	14.5 (7.2–33.4)	17.4 (8.2–31.5)	.60
Vancomycin DOT for CoNS contaminants, median (IQR)	n = 23	n = 35		n = 7	n = 20	
	4 (3–5)	3 (2–4.5)	.45	3 (2.5–4)	2 (2–3.25)	.38

The subgroup analysis excluded patients receiving antimicrobials targeted at a different infection site.

The subgroup analysis excluded patients receiving antibiotic therapy clearly targeted at another infection site, as this impacts the ability to de-escalate from broad-spectrum antimicrobials. Results were similar to the primary analysis with the additional finding that the reduction in time to anti-pseudomonal agent de-escalation reached statistical significance. These data are summarized in Table 2 and Figure 2.

A multiple linear regression model (Table 3) was created to assess the impact of BCID after adjustment for patient age, sex, ICU status, and whether the final microbial result occurred at a time with ASP support. In the primary analysis population, BCID exposure remained significantly associated with a decreased time to optimal therapy (*P* < .001) and time to anti-MRSA agent de-escalation (*P* = .039). In the secondary analysis population, BCID exposure was associated with shortened time to optimal therapy, time to anti-MRSA agent de-escalation, and time to anti-PsA agent de-escalation (all *P* < .001).

**Table 3.** Multivariable^a^ regression analysis of blood culture identification impact

	Primary analysis					
	Time to optimal therapy (n = 187)		Time to anti-MRSA de-escalation (n = 154)		Time to anti-pseudomonal de-escalation (n = 136)	
	Parameter estimate^b^ (95% CI)	*P* value	Parameter estimate (95% CI)	*P* value	Parameter estimate (95% CI)	*P* value
BCID	–24.5 (–38.7 to –10.3)	<.001	–12.5 (–24.6 to –0.4)	.043	–7.3 (–20.3 to 5.8)	.272
	Subgroup analysis^c^					
	Time to optimal therapy (n = 126)		Time to anti-MRSA de-escalation (n = 108)		Time to anti-pseudomonal de-escalation (n = 95)	
	Parameter estimate^b^ (95% CI)	*P* value	Parameter estimate (95% CI)	*P* value	Parameter estimate (95% CI)	*P* value
BCID	–24.6 (–31.8 to –17.3)	<.001	–14.6 (–21.9 to –7.4)	<.001	–17.9 (–26.1 to –9.7)	<.001

a Patient age, sex, ICU status, Charlson Comorbidity Index, and whether culture/BCID resulted during ASP availability were included as covariates.

b A negative parameter estimate indicates the parameter is associated with a decreased time to the outcome.

c The subgroup analysis excluded patients receiving antimicrobials targeted at a different infection site.

## Discussion

In this retrospective cohort study of blood cultures identified via BCID compared to those identified via standard culture-based techniques, BCID use was associated with a reduction in time to organism identification which allowed earlier initiation of optimal therapy and de-escalation of anti-MRSA agents, and a trend toward earlier anti-PsA agent de-escalation. Lack of ASP availability overnight did not significantly impact the primary outcomes. Vancomycin DOT for CoNS was numerically lower with BCID use, however this finding was not significant. These results indicate that rapid pathogen identification diagnostics allow for more optimized antimicrobial use even when implementation is restricted to daytime hours.

The magnitude of the reduction in time to optimal therapy (28.7 h) is less compared to Chiasson et al, which found a reduction in time to optimal therapy of 39.1 hours with BCID implementation (73.8 h vs 34.7 h).^3^ That study defined optimal therapy as the preferred antimicrobial agent based on evidence-based consensus guidelines, which may be more broad than our definition in which more narrow-spectrum antimicrobials were preferred. Additionally, Chiasson et al excluded polymicrobial blood cultures which may have made the studied patient population less complex to manage. Our observed reduction in time to anti-PsA agent de-escalation (16 h) is similar to previously reported reductions in piperacillin-tazobactam use of 11–12 hours.^4^ Although no significant reduction in vancomycin DOT for CoNS was detected, a numerical reduction from 4 to 3 days may be of clinical relevance.

To date, no study has demonstrated a difference in mortality associated with the use of BCID. Most critically ill patients empirically receive vancomycin in combination with an anti-pseudomonal beta-lactam meaning that rapid diagnostic testing is likely to impact mortality only when fungi or highly resistant gram-negative bacteria are detected, such as ESBL-producers or carbapenem-resistant Enterobacterales. Our study did not capture any infections caused by carbapenem-resistant Enterobacterales, and only 13 total cases of infections caused by ESBL-producers or fungi. It is therefore likely that these pathogens did not cause infection with enough frequency to impact observed mortality in this study.

Unlike previous studies, this analysis failed to demonstrate a benefit on hospital length of stay. This may be attributed to the presence of untested effect measure modifiers such as social barriers to hospital discharge (eg insurance status, lack of caregiver support) which are common at our institution, and/or the inclusion of patients with polymicrobial bloodstream infections. There were numerically more cultures with polymicrobial growth in the BCID group, which may have delayed optimal therapy or affected hospital length of stay. The median hospital length of stay for those with polymicrobial growth was 25.4 days, numerically longer than the median duration of hospital stay for both the BCID and standard groups (17.4 and 14.4 d respectively).

Use of antimicrobials targeted at another site of infection was expected to affect the ability to de-escalate from broad-spectrum antimicrobials, prevent switching to optimal therapy, and prolong hospital length of stay. In a subgroup analysis after excluding these patients, we found similar results as the primary analysis with respect to time to optimal therapy, time to anti-MRSA agent de-escalation, and all secondary outcomes. Additionally, the subgroup analysis showed a statistically significant reduction in time to anti-PsA agent de-escalation. Reduced use of broad-spectrum antimicrobials has other benefits not quantified in this study, including decreased risk of *Clostridioides difficile* infection, development of fungal and antimicrobial-resistant bacterial infections, and vancomycin associated acute kidney injury.^7–13^

Infectious diseases consultation is a potential confounder that was not assessed in this study. Expert consultation could result in quicker selection of optimal antimicrobial therapy, increased likelihood of source identification and control (ie central line removal), improved mortality, and shortened length of hospital stay.^14–17^ While not a replacement for infectious diseases consultation, ASP support does provide expert guidance on antimicrobial use. Our analysis showed that BCID use was still associated with decreased time to optimal therapy and time to de-escalation of MRSA/PsA agents when ASP support was not available. This suggests successful uptake of BCID into providers’ practice patterns despite a limited implementation schedule. Additional confounders may have been present at hours when BCID was not available (eg reduced staffing and differing clinician practices) and represent limitations of this natural experiment approach.

The ability to de-escalate based on BCID results requires confidence that these results are accurate. Thus, the eleven cases in which the BCID results were discordant with standard culture-based techniques are of interest (Table S2). Discordant cases generally fall into one of four categories: (1) failure to detect an organism on the panel (five instances), (2) failure to identify a resistance gene for an organism that was phenotypically resistant (two instances), (3) inability to differentiate between CoNS species (three instances), and (4) detection of an organism that was not identified via standard techniques (one instance).

Situations (1) and (2) represent scenarios in which failures are likely to have more severe consequences. Targeting therapy based on incorrect results in these cases results in a lack of coverage of the causative pathogen, possibly during the first 48 hours of a critical illness. Four of the five cases of BCID failing to detect an organism occurred in polymicrobial cultures. Discordance in three of these cases is attributed to growth in the opposite bottle within the set (eg aerobic bottle grew *Corynebacterium*, while the anaerobic bottle grew *B. fragilis*; BCID was run only on the aerobic bottle) and therefore not representing a true failure of the BCID, but rather a shortcoming of BCID being run on only one bottle. Of the two instances in which resistance gene results incorrectly predicted the isolate’s susceptibility, one involved an isolate of MRSA in which neither *mecA* nor *mecC* were detected. In this case, the BCID was repeated on a different bottle also growing MRSA, which again was negative for *mecA* and *mecC*. However, three days later, methicillin-sensitive *Staphylococcus aureus* (MSSA) was isolated and the consulting ID team felt this patient who used injection drugs likely had both MRSA and MSSA bacteremia. The other involved detection of *mecA* from a polymicrobial culture containing two CoNS in which both isolates were susceptible to oxacillin. Situations (3) and (4) represent scenarios in which failures are less likely to have severe consequences, resulting in unnecessary use of broad-spectrum antimicrobials or inappropriate coverage of an organism that does not need to be targeted.

This study had several limitations including the short duration and resultant small number of cultures assessed. At our institution, BCID panels are run on only the first positive bottle in a blood culture set, unless a second bottle reveals organisms with a different Gram stain morphology. This seldom resulted in organisms, most frequently suspected contaminants, being missed from BCID not being run on multiple bottles; however, these cases were still included based on correctly identifying what grew in the bottle sampled. Additionally, the underlying source of the bloodstream infection and the ability for source control were not assessed, which may affect duration of therapy and hospital length of stay. Strengths of the study include the use of a contemporaneous control group, individual chart review by at least two study investigators, and inclusion of real-world complex patients (ie polymicrobial bloodstream infections).

In summary, use of BCID was associated with significant reductions in median time to optimal therapy and median time to de-escalation of anti-MRSA agents. Additionally, BCID use was associated with a significant reduction in median time to anti-PsA agent de-escalation when controlled for antibiotics targeted at other infection sites, which has not been previously studied. These improvements were realized despite BCID being available only during daytime hours and lack of access to 24/7 ASP support. This finding has implications for the adoption of novel diagnostic technologies in other settings with limited personnel resources.

## Supporting information

Martin et al. supplementary material Martin et al. supplementary material

## Data Availability

Data not publicly available.

## Supplementary material

For supplementary material accompanying this paper visit http://doi.org/10.1017/ash.2024.51. click here to view supplementary material
