# Supplementary material for: The impact of implementation of rapid blood culture identification panels on antimicrobial optimization: a retrospective cohort study
Source: Antimicrob Steward Healthc Epidemiol. 2024 Apr 16;4(1):e44. doi: 10.1017/ash.2024.51 (PMC11019579; doi:10.1017/ash.2024.51)
Supplement: Martin et al. supplementary material [file S2732494X24000512sup001.docx]

**Supplementary Appendix**

Table S1: Organisms and antimicrobial resistance genes with targets on the Gram-positive, Gram-negative, and fungal BCID panels

| Gram-positive | Gram-negative | Fungal |
| --- | --- | --- |
| *Bacillus cereus*  *Bacillus subtilis*  *Corynebacterium spp.*  *Cutibacterium acnes*  *Enterococcus spp.*  *Enterococcus faecalis*  *Enterococcus faecium*  *Lactobacillus*  *Listeria monocytogenes*  *Micrococcus spp.*  *Staphylococcus spp.*  *Staphylococcus aureus*  *Staphylococcus epidermidis*  *Staphylococcus lugdinensis*  *Streptococcus spp.*  *Streptococcus agalactiae*  *Streptococcus anginosus*  *Streptococcus pneumoniae*  *Streptococcus pyogenes*  *mecA*  *mecC*  *vanA*  *vanB*  Pan Gram-negative^a^  Pan *Candida*^b^ | *Acinetobacter baumannii*  *Bacteroides fragilis*  *Citrobacter spp.*  *Cronobacter sakazakii*  *Enterobacter* (non-cloacae complex)  *Enterobacter cloacae* complex  *Escherichia coli*  *Fusobacterium nucleatum*  *Fusobacterium necrophorum*  *Haemophilus influenzae*  *Klebsiella oxytoca*  *Klebsiella pneumoniae*  *Morganella morganii*  *Neisseria meningitidis*  *Proteus spp.*  *Proteus mirabilis*  *Pseudomonas aeruginosa*  *Salmonella spp.*  *Serratia spp.*  *Serratia marcescens*  *Stenotrophamonas maltophilia*  CTX-M  IMP  KPC  NDM  OXA-23  OXA-48  VIM  Pan Gram-positive^c^  Pan *Candida*^b^ | *Candida albicans*  *Candida auris*  *Candida dubliniensis*  *Candida famata*  *Candida glabrata*  *Candida guilliermondii*  *Condida kefyr*  *Candida lusitaniae*  *Candida parapsilosis*  *Candida tropicalis*  *Cryptococcus gattii*  *Cryptococcus neoformans*  *Fusarium spp.*  *Rhodotorula spp.* |

^a^The pan Gram-negative target allows for the detection of *Acinetobacter spp., Bacteroides spp.,* Enterobacterales, *Neisseria spp., Pseudomonas spp.,* and *Stenotrophamonas spp.* ^b^The pan *Candida* target allows for the detection of *C. albicans, C. glabrata, C. parapsilosis,* and *C. krusei*. ^c^The pan Gram-positive target allows for the detection of *Staphylococcus spp., Streptococcus spp., Enterococcus spp., Bacillus cereus,* and *Bacillus subtilis*).

Table S2: Cases with discordance between BCID and standard identification techniques

| Number | Panel Used | BCID Result | Standard Result | Notes |
| --- | --- | --- | --- | --- |
| 1 | GP | *S. epidermidis*  *mecA* | *S. epidermidis* (methicillin-resistant)  *K. pneumoniae* (ESBL) | *K. pneumoniae* grew in the other bottle within the set |
| 2 | GP | *S. epidermidis*  *mecA* | *S. epidermidis* (methicillin-resistant)  *Corynebacterium* | *Corynebacterium* grew in the other bottle within the set |
| 3 | GP | *Corynebacterium* | *Corynebacterium*  *B. fragilis* | *B. fragilis* grew in the other bottle within the set |
| 4 | GP | No targets detected | *S. epidermidis* | Sensitivities not determined |
| 5 | GP + GN | GP:  *S. aureus*  Pan Gram-negative target  GN: *S. marcescens*  Pan Gram-positive target | *S. aureus* (methicillin-susceptibile)  *S. marcescens*  *S. consellatus* | Anaerobic bottle grew GNR and GPC in clusters, aerobic bottle grew GPC in clusters |
| 6 | GP | *S. aureus* | *S. aureus* (methicillin-resistant) | MSSA was grown on a follow up blood culture 3 days later. Likely MSSA and MRSA co-infection |
| 7 | GP | *S. epidermidis*  *mecA* | *S. epidermidis* (methicillin-sensitive)  *S. simulans* (methicillin-sensitive) | None |
| 8 | GP | *S. epidermidis* | *S. hominis* | None |
| 9 | GP | *S. epidermidis* | *S. hominis* | None |
| 10 | GP | *S. epidermidis* | *S. capitis* | None |
| 11 | GP | *Corynebacterium* | *S. epidermidis* | *S. epidermidis* grew in the other bottle within the set |

GP: Gram-positive panel; GN: Gram-negative panel; FP: Fungal panel; GNR: Gram-negative rod; GPC: Gram-positive cocci
